# The American Thyroid Association risk stratification and long-term outcomes of differentiated thyroid cancer: a 20-year follow-up of patients in Saudi Arabia

**DOI:** 10.3389/fendo.2023.1256232

**Published:** 2023-11-17

**Authors:** Anwar Ali Jammah, Ibrahim Mohammed AlSadhan, Ebtihal Y. Alyusuf, Mubarak Alajmi, Abdullah Alhamoudi, Mohammed E. Al-Sofiani

**Affiliations:** ^1^ Endocrinology and Diabetes Division, Department of Medicine, King Saud University Medical City, King Saud University, Riyadh, Saudi Arabia; ^2^ Salmaniya Medical Complex, Government Hospitals, Manama, Bahrain; ^3^ Internal Medicine Division, Department of Medicine, King Saud University Medical City, King Saud University, Riyadh, Saudi Arabia; ^4^ Endocrinology and Diabetes Division, King Saud bin Abdulaziz University for Health Sciences, Riyadh, Saudi Arabia; ^5^ Division of Endocrinology, Department of Internal Medicine, College of Medicine, King Saud University, Riyadh, Saudi Arabia; ^6^ Division of Endocrinology, Diabetes & Metabolism, The Johns Hopkins University, Baltimore, MD, United States; ^7^ Endocrinology and Diabetes Division, Strategic Center for Diabetes Research, Riyadh, Saudi Arabia

**Keywords:** thyroid cancer, outcomes, predictors, responses to treatment, Saudi Arabia

## Abstract

**Background:**

Studies have reported differing factors associated with poor outcomes in patients with differentiated thyroid cancer (DTC). We aimed to describe our 20 years of experience in the management of thyroid cancer (TC) and identify predictors of treatment outcomes.

**Methods:**

We conducted a retrospective review of medical records of patients with TC seen in the Thyroid Center at King Saud University Medical City (KSUMC) in Riyadh, Saudi Arabia, between the years 2000 and 2020. Demographic and clinical data including pathological characteristics were collected. The American Thyroid Association (ATA) risk stratification was determined for all patients at the postoperative period as well as the response to therapy at the final follow-up visit.

**Results:**

A total of 674 patients (mean age: 47.21 years) with TC, 571 (84.7%) of which were women, were included. There were 404 (60.0%) patients with ATA low risk, 127 (18.8%) with intermediate risk, and 143 (21.2%) with high-risk histology. Overall, 461 patients (68.4%) had an excellent response to treatment, 65 (9.6%) had an indeterminate response, 83 (12.3%) had a biochemical incomplete response, and 65 (9.6%) had a structural incomplete response. Patients who had an excellent response were mostly ATA low risk (*n* = 318 of 431, 68.1%), whereas 40 of 65 patients (61.5%) of those with ATA high-risk histology had a structural incomplete response to treatment. There were significantly more women who had an excellent response compared with men. Obesity, lymphovascular invasion, and size of the tumor were significant predictors of worse outcomes to therapy.

**Conclusion:**

Tumor size, lymphovascular invasion, and obesity are strong predictors of a worse response to therapy among patients with TC. Patients with obesity should be carefully followed up regardless of their risk stratification in light of the recent compelling evidence associating obesity with thyroid cancer and its higher risk of a worse disease outcome. ATA risk stratification is well correlated with patient long-term outcomes.

## Introduction

The incidence of differentiated thyroid cancer (DTC) has markedly increased during recent decades (1, 2), largely driven by the widespread use of imaging studies and fine-needle aspiration biopsies (FNAB) (3, 4),overdiagnosis, and changes in the professional guideline recommendations (5, 6). Age-standardized incidence rates were reported to be higher among women and those older than 50 years, and higher mortality has been reported among patients aged 80–84 years old (1, 3, 7).

In Saudi Arabia, thyroid cancer (TC) remains one of the most common types of cancer, especially among women aged 15–29 years old and above with an observed increase of 0.9% from 2001 to 2020 (8, 9). The incidence of TC in Saudi Arabia has increased by 3.7% since 2001 (8). Double-peak recurrence rates were observed among Saudi patients with TC at 1–2 years and 13 and 14 years from the time of diagnosis, with lower recurrence rates among patients who had received radioactive iodine therapy (RAI) (10).

The outcomes after therapy of TC vary from excellent response to structural incomplete response. Excellent response to therapy is defined as stimulated thyroglobulin (Tg) <1 ng/mL, suppressed Tg <0.1 ng/mL, and no evidence of the disease by imaging (11). Biochemical incomplete response is defined as high Tg or rising thyroglobulin antibodies following treatment without structural evidence of disease (12). TC patients with biochemical incomplete response still have a good long-term survival rate, whereas a structural incomplete response to initial therapy was associated with significantly worse clinical outcome (13, 14). Structural incomplete response to therapy, defined as having anatomical evidence of disease regardless of the Tg level and the status of anti-Tg antibodies, has worse clinical outcomes (15–17). Older age, male gender, multifocality, mediastinal or lateral lymph node (LN) involvement, and class III extent of the disease by De Groot have been identified as strong predictors of incomplete response to therapy (15, 18, 19).

Risk stratification after the initial therapy and the long-term outcome of patients with TC has been advocated for by most clinicians to prognosticate and predict the risk for persistent or recurrent disease. Data in this regard remain limited in patients with TC in the Middle East. Here, we report our experience at King Saud Medical City in Saudi Arabia and identify predictors of worse response to treatment in patients with TC.

## Materials and methods

### Study design and patients

We conducted a retrospective review of medical charts of all patients with TC diagnosed and followed up at King Saud University Medical City (KSUMC) in Riyadh, Saudi Arabia, in the last 20 years (from the year 2000 to 2020). Institutional review board approval was obtained from the KSUMC College of Medicine, Riyadh, Saudi Arabia. We excluded patients with medullary, anaplastic, and poorly differentiated TC; those without enough data for accurate staging; and those lost to follow-up.

Data collection included the demographic, clinical, and pathological characteristics; duration of follow-up after diagnosis with TC; the extent of thyroid surgery; postoperative complications; the pathological diagnosis; surgical pathology characteristics; treatment modality; baseline stimulated and non-stimulated Tg; radioactive iodine (RAI) Tg; thyroid-stimulating hormone (TSH) stimulation; TSH level; and diagnostic imaging results. Minimally invasive cancer is defined as an encapsulated follicular tumor with only small to medium vessel invasion without extension to the thyroid parenchyma. Extrathyroidal extension is defined as an extension of the tumor outside the thyroid capsule into other structures. The results of the ultrasound (US) were verified by two sonographic consultants, and the pathologic findings were verified by two consultant pathologists.

### Risk stratification of thyroid cancer and response to treatment (outcome)

Individualized risk stratification was done for all patients initially after the thyroid surgery and reassessed after 6 months to 1 year after as described by the 2009 American Thyroid Association (ATA) risk stratification as shown in the **Supplementary Table** (20). Response to therapy assessment was carried out using the dynamic risk stratification system recommended by the ATA (20). An excellent response indicates no radiological or biochemical evidence of the disease (i.e., negative imaging studies and suppressed Tg <0.1 ng/mL or stimulated Tg <1 ng/mL in the absence of anti-Tg antibodies); a biochemical incomplete response indicates suppressed Tg ≥1 ng/mL, stimulated Tg ≥10 ng/mL, or rising anti-Tg antibody levels in the absence of radiological evidence of the disease; a structural incomplete response indicates structural or functional evidence of disease with any Tg level with or without anti-Tg antibodies; and an indeterminate response was defined as having no convincing evidence of biochemical disease (i.e., suppressed Tg 0.2–1 and stimulated Tg 1.1–10 ng/mL or positive anti-Tg antibodies) or evident structural disease (20). Although the response to therapy is a dynamic assessment that was done in every follow-up visit, here, we report the results of the assessment documented in the patient’s chart from the last visit.

### Laboratory investigations

Laboratory investigations were done to measure serum TSH, free thyroxine (FT4), Tg, and anti-thyroglobulin antibodies (anti-Tg) levels. The serum concentrations of Tg, TSH, and FT4 were measured by assay on the electrochemiluminescence immunoassay (ECLIA) system (Cobas, Roche Diagnostics, Germany). The detection limit of serum Tg was 0.04–500 ng/mL, and for the TSH and FT4 assays, they were 0.005 µIU/mL and 0.023 ng/dL, respectively. The manufacturer’s reference range for TSH was 0.27–4.20 µIU/mL, and for FT4, it was 12–22 pmol/L (0.93–1.7 ng/dL). According to the manufacturer’s leaflet, the reference ranges for all thyroid parameters were determined in 2003/2004 at the Universitätsklinikum Leipzig, Leipzig, Germany, from serum specimens collected from 870 blood donors. The anti-Tg Abs were considered negative if the levels ranged from 0 to 60 units. Anti-Tg was measured by enzyme-linked immunosorbent assay (ELISA) (Inova Diagnostic, Ingbert, Germany). From 2010 to 2020, there were no changes in the assays done on the machines specified above.

### Statistical analysis

The Kolmogorov–Smirnov test was used to test the normality of data. Results were expressed as mean and standard deviation (SD) for continuous variables and as numbers and percentages for categorical variables. Comparison of mean (SD) was done using the one-way analysis of variance (ANOVA) and chi-square test to compare between categorical groups. *Post-hoc* ANOVA analysis was done to compare the mean and SD of different groups. Study participants who had an excellent response were compared with those who had a worse/unfavorable response in terms of their demographic, clinical, and pathological characteristics. A multivariate regression analysis was used to assess the association of the independent variables (gender, treatment given, age in years, size of the tumor, multiple nodules, capsular invasion, lymphovascular invasion, extranodal involvement, metastasis, and extrathyroidal extension) with the dependent variable (the outcome of treatment). The omnibus tests of model coefficients were statistically significant (*p* < 0.001), Nagelkerke *R* ^2^ = 0.143, and the Hosmer–Lemeshow test was 0.238, indicating goodness of fit. The Kaplan–Meir curves of disease outcome according to tumor histology and treatment were calculated. A *p*-value <0.05 was considered statistically significant. Data were analyzed using the Statistical Package for Social Sciences (SPSS) version 24.0 (IBM-SPSS, Armonk, New York, USA).

## Results

### Baseline clinicopathological characteristics

**Figure 1:**
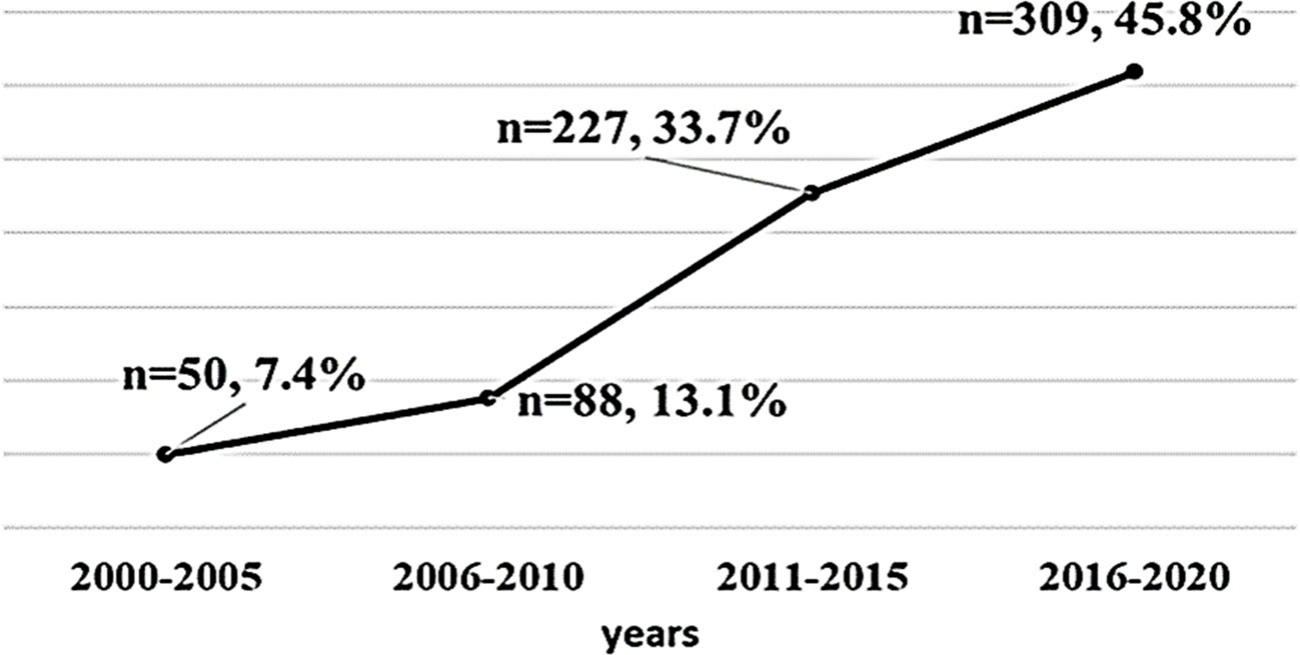
The number of DTC cases diagnosed every 5 years at our center between 2000 and 2020.

A total of 674 patients with TC were included in the study, 571 (84.7%) of which were women. The study participants had a mean age of 47.2 years (range: 14 to 88 years old), a mean duration of follow-up of 6.17 years, and a mean body mass index (BMI) at the last visit of 30.0 ± 6.5 kg/m (2). Three hundred patients had obesity (44.5%). The Bethesda classifications were as follows: 2 (0.3%) Bethesda I, 131 (19.4%) Bethesda II, 79 (11.7%) Bethesda III, 66 (9.8%) Bethesda IV,77 (11.4%) Bethesda V, 211 (31.3%) Bethesda VI, and 108 (16.0%) had no fine needle aspiration (FNA) results. Out of 674 patients with DTC, majority (*n* = 609, 90.4%) of the pathological diagnosis was papillary type, and 65 (9.6%) patients had follicular type. Based on the ATA risk stratification system, 404 (60.0%) of the patients were in the low-risk group, 127 (18.8%) in the intermediate-risk group, and 143 (21.2%) were in the high-risk group. **Table 1** shows the detailed clinicopathologic characteristics of the patients. The number of TC cases over the 20-year study period was from 7.4% in 2000–2005 to 45.8% between 2016 and 2020 (**Figure 1**).

**Table 1:** Baseline clinicopathologic characteristics of 674 patients with DTC at King Saud University Medical City, Riyadh, Saudi Arabia.

Variables	All patients (*n* = 655)
Age in years, mean ± SD	47.2 ± 13.0 (range: 14–88 years)
BMI at last visit in kg/m^2^, mean ± SD	30.0 ± 6.5
Duration of follow-up in years, mean ± SD	6.17 ± 4.7 (range: 2–20 years)
Gender, *n* (%) and age (mean ± SD)	
Men Women	103 (15.3%), 49.3 ± 14.1 571 (84.7%), 46.8 ± 12.8
BMI class at last visit, *n* (%)	
Underweight Normal Overweight Obese	10 (1.5%) 147 (21.8%) 217 (32.2%) 300 (44.5%)
Pathologic diagnosis and cell types	
Papillary thyroid carcinoma Classic Follicular Others Not specified Follicular thyroid carcinoma Minimally invasive Widely invasive Others Not specified	609 (90.4%) 224 (36.8%) 130 (21.3%) 101 (16.6%) 154 (25.3%) 65 (9.6%) 26 (40.0%) 3 (4.6%) 29 (44.6%) 7 (10.8%)
Pathological findings	
Single nodule Capsular invasion Lymphovascular invasion Presence of high-risk cells Surgical margin involvement Extrathyroidal extension Lymph node positivity Extranodal involvement	360 (53.4%) 63 (9.3%) 136 (20.2%) 28 (4.2%) 98 (14.5%) 112 (16.6%) 160 (23.7%) 30 (4.5%)
Number of positive LN, mean ± SD	2.1 ± 5.2
Number of LN examined, mean ± SD	11.8 ± 16.2
Size of the largest LN in cm, mean ± SD	1.4 ± 1.2
Size of the nodule in cm, mean ± SD	2.1 ± 1.8
Size of the lymph node in cm, mean ± SD	1.4 ± 0.8
ATA risk stratification	
Low risk Intermediate risk High risk	404 (60.0%) 127 (18.8%) 143 (21.2%)

Continuous variables are presented as mean ± SD, and categorical variables are presented as numbers and percentages.

SD, standard deviation; NIFTP, non-invasive with papillary like features; BMI, body mass index; LN, lymph node; ATA, American Thyroid Association.

### TC management

Seventy-two (10.7%) patients underwent hemithyroidectomy, whereas 602 (89.3%) patients underwent total thyroidectomy. LN dissection was performed in 176 (26.1%) patients. Following thyroidectomy, 21 (3.1%) patients had vocal cord damage and 103 (15.3%) patients had hypocalcemia up to the first postoperative year. Three-hundred and eleven (46.1%) patients received radioactive iodine ablation with a mean dose of 93.4 ± 54.6 mCi. TSH stimulation was carried out using thyrotropin in 522 (77.4%) of the patients, whereas 133 (19.7%) had thyroxine withdrawal for the same purpose. Suppressed Tg and anti-Tg levels and US neck were done for the patients routinely every 6 to 12 months, and stimulated Tg and other imaging including whole body scan (WBS), computed tomography (CT) scan, magnetic resonance imaging (MRI), and positron emission tomography (PET) scan modalities were done for the patients with high Tg or anti-Tg level according to the ATA guidelines (20).

### Response to therapy

**Figure 2:**
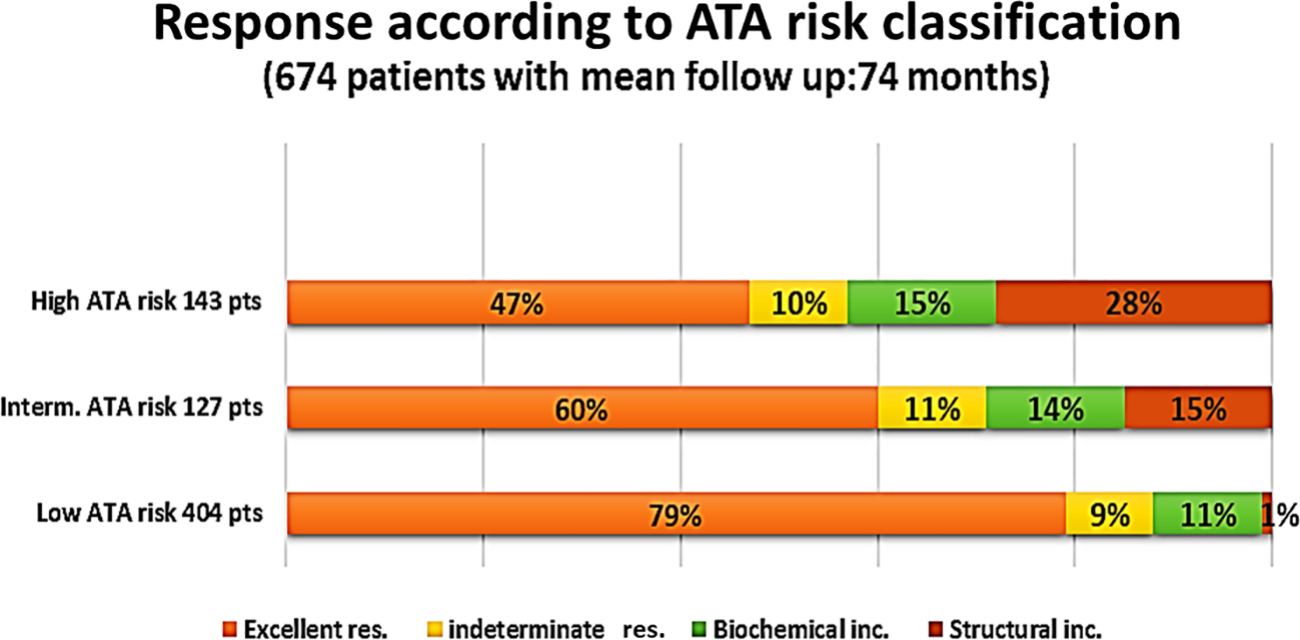
Responses according to ATA risk classification of 674 patients followed up in our center.

Overall, 461 patients (68.4%) had no evidence of the disease (excellent response), 65 (9.6%) were indeterminate cases, 83 (12.3%) had biochemical incomplete response, and 65 (9.6%) were structural incomplete response cases (**Figure 2**). The figure shows the percentage of each level of response according to the initial ATA risk stratifications. The proportion of patients with obesity was higher (*n* = 44, 67.7%) among patients who had structural incomplete response to therapy compared with patients who had excellent, indeterminate, and biochemical incomplete response to therapy (*p* = 0.025). There was no significant difference in the proportion of patients’ outcomes to therapy with regard to age (*p* = 0.621). However, ANOVA showed significant differences between the outcome to therapy and BMI at the last visit (*p* = 0.038) with *post-hoc* analysis showing a significant difference in the last visit BMI only between excellent response and structural incomplete response to therapy (*p* = 0.036). Preoperative findings of lymphovascular invasion were seen more in indeterminate, biochemical, and structural incomplete responses than with excellent response to therapy (*p* = 0.006). A higher proportion of patients with extranodal involvement were seen to have biochemical and structural incomplete responses to therapy (*p* < 0.001). The preoperative size of the cancer was significantly the largest among patients who had biochemical and structural incomplete responses to therapy (*p* = 0.003). **Table 2** shows the comparative analysis of the different variables according to the responses to therapy.

**Table 2:** Comparative analysis of the different variables according to the responses to therapy of 674 patients with DTC.

Variables	Excellent response (*n* = 461)	Indeterminate (*n* = 65)	Biochemical incomplete (*n* = 83)	Structural incomplete (*n* = 65)	*p*-values
Gender					
Men Women	9 (2.0%) 452 (98.0%)	21 (32.3%) 44 (67.7%)	20 (24.1%) 63 (75.9%)	53 (81.5%) 12 (18.5%)	0.019*
With obesity					
Yes No	216 (46.9%) 245 (53.1%)	29 (44.6%) 36 (55.4%)	33 (39.8%) 50 (60.2%)	44 (67.7%) 21 (32.3%)	0.025*
Pathologic diagnosis					
Papillary Follicular	428 (92.8%) 33 (7.2%)	53 (81.5%) 12 (18.5%)	68 (81.9%) 15 (18.1%)	60 (92.3%) 5 (7.7%)	0.003*
Age in years, mean ± SD	47.7 ± 11.9	45.8 ± 16.9	46.7 ± 14.6	46.2 ± 13.8	0.621
BMI at last visit in kg/cm^2^, mean ± SD	29.2 ± 6.2	29.9 ± 7.5	28.8 ± 6.3	31.5 ± 7.1	0.038
Duration of follow-up in years, mean ± SD	6.9 ± 4.7	3.6 ± 3.9	5.0 ± 4.1	5.2 ± 4.5	<0.001
Preoperative findings					
Lymphovascular invasion, ***n*** (%)	23 (5.0%)	42 (64.6%)	41 (49.4%)	32 (49.2%)	0.006
Surgical margins, ***n*** (%)	60 (13.0%)	11 (16.9%)	16 (19.3%)	11 (16.9%)	0.399*
Extranodal involvement, ***n*** (%)	2 (0.4%)	5 (7.7%)	12 (14.5%)	11 (16.9%)	<0.001*
Extrathyroidal extension, ***n*** (%)	12 (2.6%)	60 (92.3%)	15 (18.1%)	25 (38.5%)	0.176*
Number of positive LN, mean ± SD	1.01 ± 0.8	1.01 ± 0.8	1.22 ± 0.8	1.02 ± 0.8	0.536
Size of cancer in cm, mean ± SD	1.9 ± 1.6	2.3 ± 1.8	2.5 ± 2.6	2.6 ± 2.3	0.003
Size of the largest LN in cm, mean ± SD	1.2 ± 0.8	1.5 ± 1.4	1.6 ± 1.2	1.8 ± 1.3	0.286

*Comparative analysis of the different variables according to the responses to therapy done using the chi-square test for categorical variables. Mean and SD comparison between the different responses to therapy was done using the one-way analysis of variance (ANOVA).

Of 461 patients who had excellent response to therapy, 318 (68.9%) had low-risk histology. On the other hand, of 65 patients with structural incomplete response to treatment, 40 (61.5%) had high-risk histology (**Table 3**). Of the 65 patients who had structural incomplete disease, 30 patients (46.2%) had local (neck) disease and 19 (29.2%) were diagnosed with lung metastasis. US neck was able to detect 30 (46%) of the patients, CT neck/chest 22 (34%) of the patients, WBS 4 (6%) of the patients, and PET scan 4 (6%) of the patients.

**Table 3:** ATA risk classification according to the responses to therapy.

ATA risk classification	*n* (%)	Excellent response (*n* = 461)	Indeterminate response (*n* = 65)	Biochemicalincomplete response (*n* = 83)	Structural incomplete (*n* = 65)	*p*-values
Low risk	404 (60.0%)	318 (68.9%)	36 (55.4%)	44 (53.0%)	6 (9.2%)	<0.001
Intermediate risk	127 (18.8%)	76 (16.5%)	14 (21.5%)	18 (21.7%)	19 (29.2%)	
High risk	143 (21.2%)	67 (14.5%)	15 (23.1%)	21 (25.3%)	40 (61.5%)	

Significant differences in the proportions (p-value) were determined by chi-square analysis.

ATA, American Thyroid Association.

There were no significant differences in the mean age and duration of follow-up between men and women (49.3 ± 14.1 years versus 46.8 ± 12.8 years, *p* = 0.069; and 5.8 ± 4.4 years versus 6.2 ± 4.7 years, *p* = 0.382). TC was more common among women than men (*n* = 509 of 595 cases, 85.5%, *p* = 0.005). Furthermore, women tend to have a higher proportion of low-risk histology (62.3% versus 47.6%), whereas men have a higher tendency to have intermediate- and high-risk histology (24.3% versus 17.9 and 28.2% versus 19.7%, *p* = 0.019). More women significantly had an excellent outcome to therapy compared with men (98.0% versus 2.0%, *p* < 0.001).

### Outcome of treatment

Significant associations for structural incomplete response to therapy were found with the male gender (*p* = 0.012), obesity (*p* = 0.049), extranodal involvement (*p* < 0.001), presence of distant metastasis (*p* = 0.049), and a larger size of the tumor (*r* = 0.148, *p* < 0.001). Logistic regression analysis revealed obesity [odds ratio (OR) = 1.885, 95% CI: 1.855 to 1.915, *p* < 0.001], lymphovascular invasion (OR = 1.887, 95% CI = 1.072 to 3.662, *p* = 0.050), and the size of the tumor (OR = 1.158, 95% CI: 1.011 to 1.327, *p* = 0.034) as the significant predictive factors for structural incomplete response to therapy. Gender, tumor histology, extrathyroidal extension, and extranodal involvement were not statistically significant predictive factors for structural incomplete response to therapy (**Table 4**).

**Table 4:** Logistic regression analysis of predictors of structural incomplete response to therapy in 674 patients with DTC.

Predictors	OR	95% CI	*p*-values
Male gender	1.714	0.842 to 3.488	0.137
Obesity	1.885	1.855 to 1.915	<0.001
Pathologic diagnosis	1.197	0.455 to 3.150	0.716
Lymphovascular invasion	1.887	1.072 to 3.662	0.050
Size of tumor	1.158	1.011 to 1.327	0.034
Extrathyroidal extension	0.013	−0.018 to 0.044	0.398
Extranodal involvement	0.656	0.470 to 7.912	0.362

The model for the logistic regression analysis included the significant variables that were associated with structural incomplete response to therapy.

OR, odds ratio; CI, confidence interval.

## Discussion

TC is the most common endocrine malignancy with an increasing prevalence worldwide (1–4). A 2020 report from Saudi Arabia showed a TC prevalence rate of 12.9% with OR = 6.77 (2.34–19.53) (21). This current study showed a steep rise from 7.4% to 45.8% over the last 20 years, 90.4% of which were of the papillary type. This is consistent with earlier reports from both local and international studies (3, 10, 22). Many believe that the papillary type has favorable survival rates. However, patients are confronted with misperceptions and mixed emotions, particularly in their fight toward embattling the disease (23). The financial impact on thyroid cancer survivors reports greater psychological financial hardship than non-thyroid cancer survivors (24). There is also the issue of overdiagnosis and overtreatment of low-risk thyroid cancer, where physicians perform inappropriate biopsies and radioactive iodine treatment (25).

Women in the 40–50 age group seemed more vulnerable, which is consistent with previous studies from Saudi Arabia where TC has a significant preponderance to the female gender (8, 9). However, the recurrence rate and the proportion of a more advanced disease are higher among men (26). In this study, 24.3% and 28.2% of men with TC had intermediate- and high-risk histology compared with 17.9% and 19.7% of women. A higher proportion of men had extranodal involvement and distant metastasis despite both genders having no significant difference in the treatment received. This is consistent with the report of Zahedi et al. in 2020 and other previous studies that men tend to present with more advanced diseases (26–28).

We also found that the size of cancer >3 cm is an independent risk factor for the persistence of the disease, concordant with reports from previous studies (29–31). A larger tumor size may have an impact on the relative risk of not being cured (29). Lymphovascular invasion has been associated with disease recurrence and compromised survival in patients with thyroid cancer. Tumor size has been found to be positively associated with the risk of lymphovascular invasion, particularly in the papillary type of TC (29–31). The same goes with extrathyroidal extension since patients are considered to be in the advanced stage of the tumor (32).

One interesting finding in this study is the significant association of obesity with structural incomplete response to therapy, thus a higher risk of worse outcomes validating reports from previous studies (4, 33–37). Obesity has been proposed as a risk factor for several diseases including increased risk for TC. In many studies, women who were diagnosed with TC had a higher prevalence of obesity with higher waist circumference and higher adipose ratio (4, 33). The OR reported from other countries was 1.63 per 5 kg/m (2) increase in BMI (4, 33–36). One article reported that in one of every six TCs diagnosed among adults 60 years or older, nearly two-thirds of the patients were overweight to obese (37). According to Schmid et al., a 0.1-point rise in the waist-to-hip ratio and a five-point increase in BMI each may increase the risk of thyroid cancer by 30% and 14%, respectively (38). According to reports, there are a number of factors that raise the risk of thyroid cancer in those who are obese. The risk for the development of thyroid cancer may increase if aromatase levels are raised and activated because of an imbalance in estrogen and androgen hormones (39, 40). Another potential mechanism is an upsurge in oxidative stress brought on by immune system non-specific activation and inflammatory substances like cytokines and adipokines that lead to an excess of reactive oxygen species (ROS) (39–43). Recent studies have consistently found evidence that obesity raises the risk for thyroid cancer (39–43). However, a precise molecular mechanism underlying the association between obesity and TC needs to be identified. Clinicians should closely monitor patients, regardless of their initial risk categorization, in light of the recent compelling evidence associating obesity with thyroid cancer and its higher risk of a worse disease outcome. Additional clinical evaluations should be carried out to frequently check the thyroid function of obese patients.

The diagnostic performance of the different imaging procedures revealed that the US of the neck was positive in 63.8% of the patients who ultimately had a structural incomplete response to treatment. This implies that US offers a high sensitivity and positive predictive value for TC screening (44, 45). In addition, preoperative ultrasound may change the intended surgical strategy (46, 47). According to a meta-analysis, CT exhibited an overall sensitivity of 62% and a specificity of 87% for detecting cervical lymph node metastases in TC patients (48, 49).

This study showed a structural incomplete response to treatment in 29.2% to 61.5% of patients with moderate- to high-risk histology according to the ATA risk stratification and 9.2% among those with low risk, consistent with previous studies (18, 50). Both for intermediate-risk and high-risk groups, the ATA risk class demonstrated to be a strong and credible predictor of structural persistent disease (51). We followed our patients for a longer period of time (20 years) compared with the study of Grani et al. (51) This indicates that even for long-term prediction of TC treatment results, the ATA risk stratification’s dependability is consistent. Additionally, data demonstrate that ATA risk stratification adequately classifies patients based on a variety of crucial clinical outcomes (50–55).

Many authors have proposed various characteristics that are associated with disease-free survival (DFS), including primary tumor size, multifocality, vascular invasion, extrathyroid extension, lymphovascular invasion, and lymph node ratio (50–59). In line with earlier research, extranodal involvement, lymphovascular invasion, and initial tumor size—particularly those >3 cm in size—were the most important predictors of a structural incomplete response to therapy. Patients with high-risk histology who underwent surgery and then RAI have the best prognoses and have high chances of disease-free survival (56, 60, 61). Consistent with the findings from earlier studies, we found that patients with TC treated with surgery and RAI had significantly longer recurrence-free survival times compared with patients with the papillary type treated with surgery alone and also longer recurrence-free survival times compared with patients with the follicular type treated with surgery alone or with RAI (60–63).

The extensive 20-year follow-up period is a major strength of our study. Moreover, we have studied a larger number of patients (*n* = 674) than those reported by other studies (20). However, our study has several limitations, including the retrospective nature of the study that limited us to identify several potential predictors as well as the assessment of disease-specific mortality. Furthermore, the study spanned 20 years of follow-up, in which treatment time and surgical strategies have significantly changed and probably also the number of patients with obesity. Also, the gender disparity between excellent response and structural incomplete response may have raised concern about possible selection biases since most of our patients were women. Other potential risk factors for the course of the disease, such as family history, the environment, and lifestyle choices, were not documented.

## Conclusion

The size of cancer, lymphovascular invasion, and obesity are strong predictors of a structural incomplete response to therapy among patients with TC. Patients with obesity should be carefully followed up regardless of the risk stratification in light of the recent compelling evidence associating obesity with thyroid cancer and its higher risk of a worse disease outcome. ATA risk stratification is well correlated with patient long-term outcomes and the type of response and can help to determine the initial type of surgery and radioactive iodine as well as the follow-up plan.

## Data availability statement

The raw data supporting the conclusions of this article will be made available by the authors, without undue reservation.

## Ethics statement

The studies involving humans were approved by the King Saud University Medical City College of Medicine IRB Board, Riyadh, Saudi Arabia. The studies were conducted in accordance with the local legislation and institutional requirements. The participants provided their written informed consent to participate in this study.

## Author contributions

AJ: Conceptualization, Formal Analysis, Methodology, Project administration, Supervision, Writing – original draft, Writing – review & editing. IA: Data curation, Investigation, Methodology, Resources, Writing – review & editing. EA: Data curation, Formal Analysis, Methodology, Writing – review & editing. MA: Data curation, Investigation, Methodology, Resources, Writing – review & editing. AA: Formal Analysis, Investigation, Methodology, Software, Writing – review & editing. MA-S: Conceptualization, Data curation, Investigation, Methodology, Resources, Validation, Visualization, Writing – review & editing.
