# Supplementary material for: The American Thyroid Association risk stratification and long-term outcomes of differentiated thyroid cancer: a 20-year follow-up of patients in Saudi Arabia
Source: Front Endocrinol (Lausanne). 2023 Nov 17;14:1256232. doi: 10.3389/fendo.2023.1256232 (PMC10690932; doi:10.3389/fendo.2023.1256232)
Supplement: Supplementary file 1 [file Table_1.docx]

Supplementary Table. ATA 2015 Risk stratification system with proposed modifications

| Risk | Description |
| --- | --- |
| ATA low risk | Papillary thyroid cancer (with all the following):   - No local or distant metastases - All macroscopic tumor has been resected - No tumor invasion of loco-regional tissues or structures - The tumor does not have aggressive histology - If I^13^1 is given, there are no RAI-avid metastatic foci outside the thyroid bed on the first postponement whole body RAI scan - No vascular invasion - Clinical N0 or ≤5 pathologic N1 micromestastases (<0.2 cm in largest dimension) - Intrathyroidal, encapsulated follicular variant of PTC - Intrathyroidal, well-differentiated FTC with capsular invasion and no or minimal (<4 foci) vascular invasion - Intrathyroidal, papillary microcarcinoma, unifocal or multifocal, including BRAFV*^600E^* mutated (if known) |
| ATA intermediate risk | Microscopic invasion of tumor into the perithyroidal soft tissues  RAI-avid metastatic foci in the neck on the first posttreatment whole- body RAI scan  Aggressive histology (e.g. tall cell, hobnail variant, columnar cell carcinoma)  PTC with vascular invasion  Clinical N1 or >5 pathologic N1 with all involved lymph nodes <3 cm in largest dimension  Multifocal papillary microcarcinoma with ETE and BRAF*^V600E^* mutated (if known) |
| ATA high risk | Macroscopic invasion of tumor into the perithyroidal soft tissues (gross ETE)  Incomplete tumor resection  Distant metastases  Postoperative serum Tg suggestive of distant metastases  Pathologic N1 with any metastatic lymph node ≥3 cm in largest dimension  FTC with extensive vascular invasion (>4 foci of vascular invasion) |

Source: ^21^ Haugen BR, Alexander EK, Bible KC, Doherty GM, Mandel SJ, Nikiforov YE, Pacini F, Randolph GW, Sawka AM, Schlumberger M, Schuff KG. 2015 American Thyroid Association management guidelines for adult patients with thyroid nodules and differentiated thyroid cancer: the American Thyroid Association guidelines task force on thyroid nodules and differentiated thyroid cancer. Thyroid. 2016 Jan 1;26(1):1-133.
